# Comparative efficacy and safety of different traditional Chinese medicine external therapies for polycystic ovary syndrome in women: A network meta-analysis

**DOI:** 10.1097/MD.0000000000044441

**Published:** 2025-10-24

**Authors:** Jiahui Ye, Hua Guo, Qianru Zeng, Ziqing Gan, Qilin Jin, Hong Su, Hanmei Lin

**Affiliations:** a Postgraduate Education, Guangxi University of Chinese Medicine, Nanning, Guangxi Zhuang Autonomous Region, China; b Department of Gynecology, The First Affiliated Hospital of Guangxi University of Chinese Medicine, Nanning, Guangxi Zhuang Autonomous Region, China; c College of International Education, Guangxi University of Chinese Medicine, Nanning, Guangxi Zhuang Autonomous Region, China.

**Keywords:** external treatment of traditional Chinese medicine, network meta-analysis, PCOS, systematic review

## Abstract

**Background:**

Polycystic ovary syndrome (PCOS) is one of the most common endocrine diseases in women. In recent years, traditional Chinese medicine (TCM) treatment has had a positive effect on PCOS, and it is simple, convenient, and inexpensive. The purpose of this study was to compare the efficacy and safety of different external treatments in the treatment of PCOS using a network meta-analysis method.

**Methods:**

The databases examined comprised Web of Science, Embase, PubMed, The Cochrane Library, China National Knowledge Infrastructure, VIP, Wanfang Data Knowledge Service Platform, and the China Biomedical Literature Database. Since the establishment of the database, randomized controlled trials related to TCM external treatment for PCOS have been screened. The literature was derived from follicle-stimulating hormone (FSH), luteinizing hormone (LH), LH/FSH ratios, body mass index, and testosterone, with detrimental impacts identified as the primary outcomes of the screening process. Odds ratio values with 95% confidence intervals and standardized mean difference values were used as performance measures to compare the effects of different interventions and rank them. The above data were statistically analyzed using STATA 17.0 software.

**Results:**

Electroacupuncture has the highest cumulative probability of increasing FSH levels (surface under the cumulative ranking curve [SUCRA]: 86%) and is considered the best intervention to improve FSH in patients with PCOS. Regulating the conception-governor vessel had the highest cumulative probability of reducing LH levels (SUCRA: 76.6%), making it the best choice for improving LH in PCOS patients. Abdominal acupuncture has the highest cumulative probability (SUCRA: 58.6%) and is therefore the best intervention to improve LH/FSH in patients with PCOS. It also showed the best intervention effect with the highest cumulative probability of reducing testosterone levels (SUCRA: 98.2%). Acupuncture + medication had the highest cumulative probability of reducing body mass index (SUCRA: 98.2%) and was considered the most effective intervention. At the same time, abdominal acupuncture had the highest cumulative probability of adverse effects (SUCRA: 58.6%), indicating that it may be associated with adverse events.

**Conclusion:**

Different TCM external treatments benefit various PCOS-related indicators. Electroacupuncture, regulating conception-governor vessel, abdominal acupuncture, and acupuncture combined with drugs each excel in specific outcomes. However, abdominal acupuncture may carry a higher risk of adverse events.

## 1. Introduction

Polycystic ovarian syndrome (PCOS) has emerged as a significant public health concern worldwide, with its estimated prevalence varying between 5% and 15%.^[1]^ The condition is primarily defined by 3 hallmark features: menstrual dysfunction, clinical or biochemical hyperandrogenism, and the presence of polycystic ovarian morphology on imaging. Insulin resistance (IR) and high androgen levels are considered key factors in the pathogenesis and progression of PCOS and play an important role in its pathological mechanisms.^[2,3]^ Studies have found that PCOS is not only a major cause of anovulatory infertility but also affects about 2% to 40% of women of childbearing age.^[4]^ Moreover, PCOS serves as a significant predisposing factor for multiple metabolic disorders, including type 2 diabetes, cardiovascular disease, and endometrial cancer.^[5–7]^ In addition, women with PCOS are more likely to experience depression and anxiety than the average woman.^[8]^ In the United States, the economic burden of PCOS was estimated to be as high as $8 billion per year in 2020,^[9]^ showing the enormous economic and health pressures it places on society and individuals.

Managing PCOS continues to present substantial clinical challenges, as existing therapeutic strategies are primarily aimed at alleviating symptoms rather than targeting the fundamental pathophysiological mechanisms of the disorder.^[10]^ To date, no medications have received specific approval from the U.S. Food and Drug Administration for the treatment of PCOS. First-line treatment drugs usually have the primary goal of inducing ovulation to restore a woman’s fertility. Although drugs such as aromatase preparations, oral contraceptives, antiandrogens, and ovulation-inducing drugs have made some progress in improving PCOS symptoms, they still have certain limitations, such as clinical resistance, nausea, ovarian hyperstimulation syndrome, and other side effects, especially in long-term treatment.^[11]^ The 2023 International Evidence-Based Guideline for the Assessment and Management of PCOS continues to recommend metformin as a first-line agent for improving IR in women with PCOS.^[12]^ Studies have shown that short-term use of metformin at a daily dose of 1500 mg for 3 to 4 months significantly reduces levels of luteinizing hormone (LH), follicle-stimulating hormone (FSH), fasting insulin, homeostasis model assessment of IR, blood pressure, waist circumference, and serum testosterone compared to placebo.^[13]^ In addition, early pregnancy use of metformin in women with PCOS has been associated with a significant reduction in the risk of early miscarriage.^[14]^ Nevertheless, the main adverse effects of metformin are gastrointestinal discomfort, such as diarrhea and abdominal pain, along with vitamin B12 deficiency during long-term use. In rare cases, metformin-associated lactic acidosis may occur, particularly in individuals with severe renal impairment, which constitutes a major contraindication to its use.^[15]^ Consequently, there is an imperative necessity to formulate noninvasive, symptom-focused preventive treatment alternatives to more effectively meet the therapeutic requirements of PCOS.

In East Asia, traditional Chinese medicine (TCM) external treatment is widely regarded as an effective complementary treatment for PCOS. In East Asia, TCM external treatment is widely regarded as an effective complementary treatment for PCOS.^[16,17]^ For example, auriculotherapy, as a simple, inexpensive, noninvasive, and straightforward treatment, has attracted more and more attention in recent years.^[18]^ Evidence suggests that auriculotherapy may contribute to reductions in body mass index (BMI) among patients with PCOS.^[19]^ Additionally, acupuncture has been demonstrated to modulate sex hormone levels, facilitate weight loss, and decrease serum anti-Müllerian hormone concentrations in this population.^[20]^ Compared to metformin, acupuncture appears to exert superior effects on glucose metabolism, potentially reducing the risk of developing type 2 diabetes, and is associated with fewer adverse effects.^[21]^ Moreover, acupuncture and moxibustion-related therapies, when used as complementary or alternative treatments, may enhance the therapeutic efficacy of conventional pharmacological interventions.^[22]^ The combination of acupuncture and pharmacological treatment has shown greater efficacy in improving pregnancy outcomes and significantly increasing pregnancy success rates compared to drug therapy alone in patients with PCOS.^[23]^ A separate meta-analysis further indicated that acupuncture combined with metformin enhanced ovulation rates, pregnancy outcomes, and IR more effectively than metformin monotherapy.^[24]^ The therapeutic mechanisms of acupuncture in PCOS primarily involve modulation of the hypothalamic-pituitary-ovarian (HPO) axis,^[25]^ regulation of the PI3K/Akt/mTOR autophagy signaling pathway,^[26]^ and restoration of gut microbiota balance,^[27]^ reflecting its potential for multi-targeted intervention.

Although multiple meta-analyses of TCM external treatments have shown their efficacy in the treatment of PCOS,^[22,24,28,29]^ due to the wide variety of external treatments in TCM and differences in efficacy, the limitations of traditional meta-analysis methods are obvious. Traditional methods can only make direct comparisons between single therapies, making it difficult to comprehensively assess the relative effects of multiple therapies. By integrating direct and indirect evidence, a network meta-analysis (NMA) is able to systematically compare and rank the efficacy of multiple treatments to identify the optimal treatment options.^[30]^ This study therefore, gathered randomized controlled trials (RCTs) of TCM external treatment for PCOS, both domestically and internationally, in recent years and employed NMA methodologies to identify optimal treatment alternatives, thereby offering robust evidence-based support for clinical practice.

## 2. Methods

### 2.1. Study registration

The protocol for this systematic review was registered in the International Prospective Register of Systematic Reviews (PROSPERO, CRD42024567890) on February 23, 2025.

### 2.2. Inclusion criteria

1. All RCTs of TCM external treatment for PCOS will be included.
2. Eligible studies must meet the diagnostic criteria of the 2023 International Evidence-Based Guidelines for PCOS.^[12]^
3. The experimental group received external treatments of TCM, either alone or in combination with conventional pharmacological therapy or standard clinical care. These TCM external therapies included acupuncture (the standardized practice of inserting fine needles into defined acupuncture points [acupoints] based on TCM theory, often aimed at regulating qi, alleviating pain, or treating disease), electroacupuncture (EA, a method that applies low-frequency electrical stimulation through acupuncture needles), warm acupuncture (acupuncture combined with heat stimulation, typically using moxa attached to the needle), moxibustion (the burning of dried mugwort near or on acupuncture points to stimulate circulation and energy flow), acupoint application (the topical application of herbal pastes or medicated patches on specific acupuncture points), acupoint massage (manual stimulation of acupuncture points through pressing or kneading), auricular point therapy or auriculotherapy (stimulating specific points on the external ear that are believed to correspond to internal organs), tuina (a traditional Chinese manual therapy involving pressing, rubbing, and stretching techniques), and general massage (soft tissue manipulation aimed at relaxing muscles and promoting circulation). The control group received either standard pharmacological treatment, sham acupuncture (which involves superficial needling [<5 mm] at non-acupoints, stimulation of pseudoacupoints located outside traditional meridian lines without manual manipulation, or the use of non-penetrating sham acupuncture devices) or a different form of TCM external therapy in combination with conventional medical treatment.
4. Primary outcomes were consistent, including but not limited to FSH, LH, LH/FSH, BMI, testosterone (T), and adverse effects.

### 2.3. Exclusion criteria

1. Duplicate publications or duplicate data.
2. Literature with incomplete data.
3. The types of studies excluded were reviews, systematic reviews, meta-analyses, cross-sectional studies, case reports, cohort studies, and animal experiments.
4. Based on the same conventional Western medicine treatment for the underlying disease, the control group patients were treated with the same Western medicine treatment as the experimental group.

### 2.4. Search strategy

All RCTs of PCOS were limited to English and Chinese. The Chinese databases searched included China National Knowledge Infrastructure, Wanfang Data Knowledge Service Platform, VIP Chinese Journals Full-text Database, and China Biomedical Literature Database.

Searches of English databases included PubMed, Web of Science, Embase, and The Cochrane Library. In the search strategy, the following terms were used: [(“polycystic ovary syndrome”) AND (“external therapy” OR “acupuncture” OR “needling” OR “moxibustion” OR “electroacupuncture” OR “manipulation” OR “massage” OR “acupoint application” OR “acupoint sticking” OR “acupoint injection” OR “auricular point” OR “scraping therapy” OR “external treatment of TCM” OR “TCM therapy”)]. The search is current as of December 11, 2024.

Two authors independently screened the titles and abstracts of the retrieved articles. Duplicates and irrelevant studies were excluded, and the final inclusion or exclusion decision was made through consensus, based on predefined inclusion criteria. One author extracted the relevant data, including basic study information, objectives, results, and follow-up data, which was subsequently reviewed by the second author. In the event of a disagreement, a third-party advisor made the final decision.

### 2.5. Data extraction

Two researchers worked independently on the literature screening and data extraction, cross-checked the results, and resolved conflicts through negotiation with a third researcher. Initially, the retrieved studies were imported into EndNote 21 for duplicate record removal. Next, studies clearly inconsistent with the inclusion and exclusion criteria were excluded by reviewing titles and abstracts. The full texts of the remaining studies were then downloaded and evaluated to identify eligible studies. A standardized data extraction table was created, with the following key information extracted: basic study details, including the first author, year of publication, sample size, and characteristics of the study population (gender, age, and disease duration); interventions, including treatment methods, duration, and the number of completed cases in both the experimental and control groups; outcome measures, including FSH, LH, LH/FSH, T, BMI, and adverse effects.

### 2.6. Risk of bias assessment

The Cochrane systematic review risk of bias assessment tool was adopted to assess the quality of the literature.^[31]^ Two researchers independently evaluated the risk of bias in the included studies and consulted a third researcher to resolve any disagreements. The risk of bias assessment tool consisted of 7 criteria: random sequence generation, allocation concealment, blinding of investigators and participants, blinding of outcome assessors, completeness of outcome data, selective reporting of outcomes, and other potential biases. Each criterion was assessed with one of 3 ratings: “no,” “unclear,” or “yes,” indicating high, unclear, or low risk of bias, respectively.

### 2.7. Data analysis

Heterogeneity was assessed using *I*^2^ and the *P*-value. When *P* ≥ .05 and *I*^2^ ≤ 50%, indicating no statistical heterogeneity, the fixed-effect model was used to pool the effect size. When *P* < .05 and *I*^2^ > 50%, indicating significant heterogeneity, subgroup analysis, sensitivity analysis, and other methods were used to explore the sources of heterogeneity. If the source of heterogeneity could not be ruled out, the random-effects model was used. Evidence diagrams were used to describe the relationships between interventions. The inconsistency model was used to perform a global inconsistency test, where *P* < .05 indicated inconsistency, while *P* ≥ .05 indicated consistency. A local inconsistency test was performed using the node-splitting method, where *P* < .05 indicated local inconsistency, while *P* ≥ .05 indicated local consistency. A loop inconsistency test was conducted in the presence of closed loops, and the inconsistency factor with a 95% confidence interval containing 0 indicated agreement between direct and indirect evidence. Interventions were rated based on the probability of the area under the cumulative ranking curve (surface under the cumulative ranking curve [SUCRA]), with a greater SUCRA value indicating a higher ranking on this efficacy metric and implying that it may be the best treatment option. A comparative-adjusted funnel plot was plotted to identify publication bias.

## 3. Results

### 3.1. Literature search results

**Figure 1.**
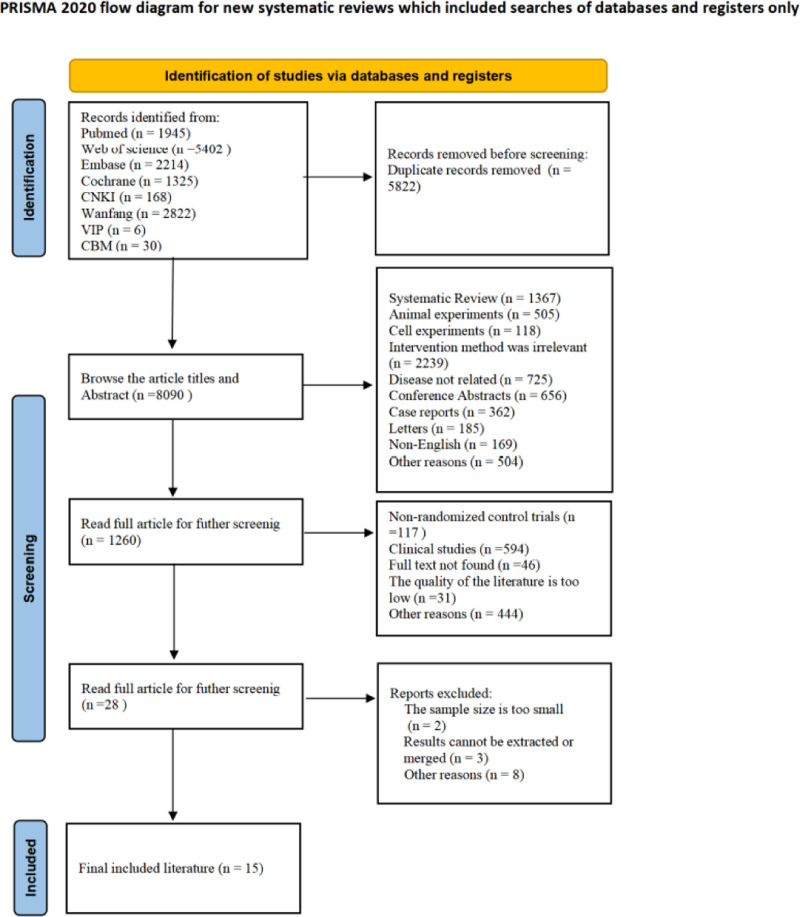
Flowchart of literature retrieval and screening. CBM = China Biomedical Literature Database, CNKI = China National Knowledge Infrastructure. Adapted from information publicly available on the PROSPERO database (International Prospective Register of Systematic Reviews, University of York).

A total of 13,912 publications were obtained in the preliminary literature search, with 5822 duplicate studies eliminated. Article titles and abstracts were scrutinized according to inclusion and exclusion criteria, resulting in the exclusion of 8090 studies, while 28 potentially relevant studies were initially included. After analyzing the full text and supplementary material, 15 RCTs^[21,32–45]^ were included for data extraction and statistical analysis. The flowchart of literature retrieval and screening is shown in Figure 1.

### 3.2. Basic characteristics of the included literature

In 15 RCTs, including 1274 patients with POCS, 11 different treatment regimens were used. In these regimens, more patients were treated with acupuncture (Table 1). The studies were published between 2010 and 2022.

**Table 1:** Clinical and demographic characteristics of studies included in the meta-analysis.

Study	Year	Country	Study type	Intervention mode		Number of cases			Age (yr)			Duration (mo)			Treatment (d)		Outcome
				Test group	Control group	Test group		Control group	Test group		Control group	Test group		Control group				
Xu and Zuo^[32]^	2018	China	RCT	Acupuncture + Medication	Medication	30		30	25.7 ± 4.0		25.8 ± 4.2	2.8 ± 2.7		3.0 ± 2.7	60		BMI, FSH, LH, T, Adverse	
Ma et al^[33]^	2016	China	RCT	FNT + Medication	Medication	30		30	23.0 ± 2.0		25.0 ± 4.0	48.6 ± 16		48.5 ± 13.6	90		T, Adverse	
				FNT		30			24.0 ± 3			52.7 ± 19						
Cao et al^[34]^	2017	China	RCT	Acupuncture	Medication	27		25	31 ± 3		29 ± 5	40 (60)		37 (33)	84		BMI, FSH, LH, LH/FSH, T, Adverse	
Yu et al^[35]^	2018	China	RCT	EA + Medication	Medication	40		40	30 ± 4		29 ± 5	3.6 ± 2.6		3.4 ± 2.3	90		Adverse	
Zhuo et al^[36]^	2016	China	RCT	RCGV	Medication	50		50	29 ± 5		28 ± 5	3.63 ± 1.68		3.76 ± 1.75	90		FSH, LH, T	
Jedel et al^[37]^	2010	Sweden	RCT	EA	Control	29		15	29.7 ± 4.3		30.1 ± 4.2	NA		NA			FSH, LH, LH/FSH, T	
				Physical Exercise		30			30.2 ± 4.7						32			
Yang et al^[38]^	2017	China	RCT	Acupuncture	Medication	29		29	27 ± 5		27 ± 3	4.1 ± 1.8		4.0 ± 1.8	90		BMI, FSH, LH, LH/FSH, T, Adverse	
Johansson et al^[39]^	2013	Sweden	RCT	Acupuncture	Attention control	16		12	27.9 ± 3.2		28.4 ± 3.1	NA		NA	90		BMI, FSH, LH, T	
Zhao et al^[40]^	2021	China	RCT	EA + Medication	Medication	30		30	27 ± 5		30 ± 6	6 ± 4		6 ± 5	84		FSH, LH, T, Adverse	
Zheng et al^[41]^	2013	China	RCT	Abdominal acupuncture	Medication	43		43	26.5 ± 3.0		24.9 ± 4.9	3.5 ± 2.8		3.6 ± 2.2	180		BMI, LH, FSH, T, LH/FSH, Adverse	
Wen et al^[21]^	2022	China	RCT	Acupuncture/metformin	Sham acupuncture	114		114	27.0 (25.0–31.0)/27.0 (25.0–30.0)		27.0 (24.0–29.0)	NA		NA	90		BMI, LH, FSH, LH/FSH, T, Adverse	
Dong et al^[42]^	2022	China	RCT	Acupuncture	Sham acupuncture	27		27	23.3 ± 2.7		22.3 ± 2.4	10.1 ± 6.6		9.6 ± 6.8	NA		BMI, LH, FSH, LH/FSH, T, Adverse	
Pastore et al^[43]^	2011	China	RCT	Acupuncture	Sham acupuncture	40		44	28.0 ± 6.3		26.5 ± 5.8	NA		NA	56		BMI, LH, FSH, Adverse	
Cao et al^[44]^	2019	China	RCT	Acupuncture	CPA/EE	30		30	29.2 ± 4.1		28.5 ± 4.2	58.8 ± 54		36.1 ± 25	168		BMI, FSH, LH, LH/FSH, T, Adverse	
Dong et al^[45]^	2021	China	RCT	EA	Sham EA	26		20	23.3 ± 2.7		22.3 ± 2.4	NA		NA	112		BMI	

BMI = body mass index, CPA/EE = cyproterone acetate/ethinylestradiol, EA = electroacupuncture, FNT = flying needling therapy, FSH = follicle stimulating hormone, LH = luteinizing hormone, RCGV = regulating conception-governor vessel, RCT = randomized controlled trial, T = testosterone. Adapted from information publicly available on the PROSPERO database (International Prospective Register of Systematic Reviews, University of York).

### 3.3. Included in the quality assessment of studies

**Figure 2.**
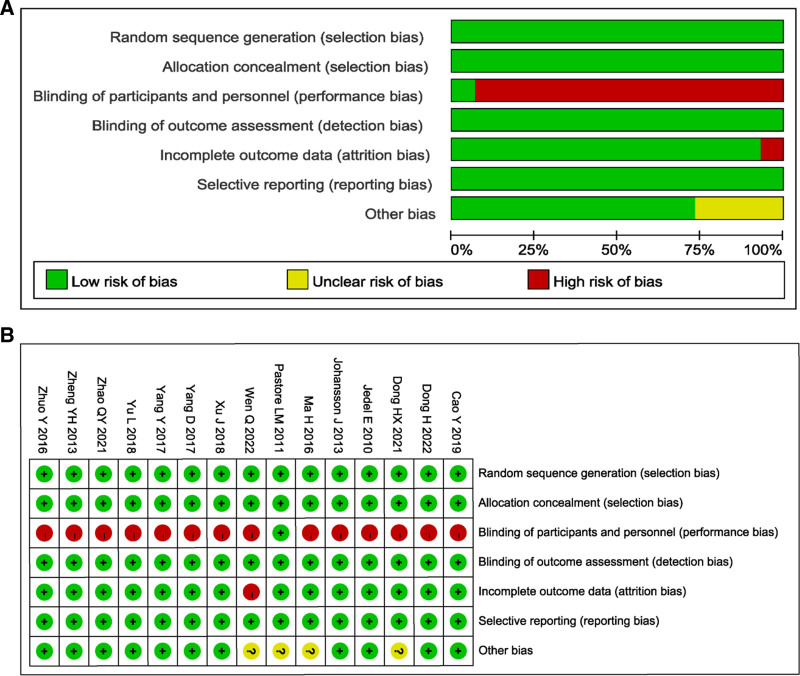
Literature quality evaluation.

The risk of bias assessment for the 15 included studies is presented in Figure 2. Regarding random sequence generation and allocation concealment, all studies were judged to have a low risk of bias (100%). In terms of blinding of participants and personnel, most studies were assessed as having a high risk of bias, except for Pastore et al^[43]^, which was rated as low risk. For blinding of outcome assessment, all studies demonstrated a low risk of bias. Concerning incomplete outcome data, the majority of studies showed a low risk of bias, with the exception of Wen et al^[21]^, which was rated as high risk. In the domain of selective reporting, all studies were considered to have a low risk of bias, with no apparent concerns identified. Regarding other sources of bias, most studies were judged to have a low risk, although Wen et al^[21]^, Pastore et al^[43]^, Ma et al^[33]^, and Dong et al^[45]^ were assessed as having an unclear risk. Overall, the included studies exhibited a generally low risk of bias.

### 3.4. Network meta-analysis

The 15 included studies covered: abdominal acupuncture, acupuncture, Dong’s extraordinary acupoints (DEA), EA, flying needling therapy (FNT), Physical Therapist, and regulating conception-governor vessel (RCGV) (7 TCM external treatments).

### 3.5. Follicle-stimulating hormone

**Figure 3.**
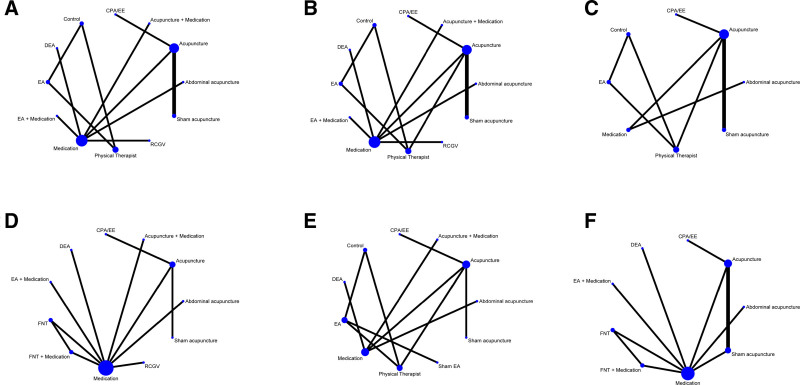
Network graph.

**Figure 4.**
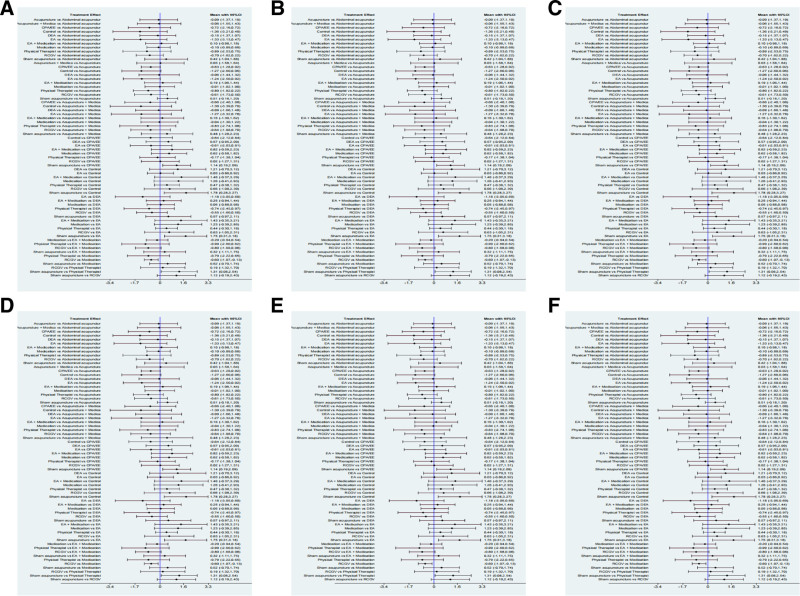
Comparison of forest plots.

Eleven studies^[32,34,36–44]^ reported FSH, and the evidence network is typically centered around acupuncture (Fig. 3A), which contains 12 nodes. Acupuncture was the most common experimental group, and medication was the most common control group. The NMA generated 66 direct or indirect comparisons, 5 of which were statistically significant, while the remaining were not, as shown in Figure 4A. SUCRA results show that, in terms of ranking probability, EA has the highest cumulative probability (SUCRA: 86%), making it the best intervention to improve FSH in patients with PCOS (Figure S1A, Supplemental Digital Content, https://links.lww.com/MD/Q404). The ranking order is as follows: EA (86%) > Control (85.9%) > RCGV (70.2%) > Physical Therapist (69%) > cyproterone acetate/ethinylestradiol (CPA/EE) (67.2%) > DEA (40.5%) > Medication (36.7%) > Acupuncture + Medication (36.5%) > Acupuncture (35.6%) > Abdominal Acupuncture (32%) > EA + Medication (26.5%) > Sham Acupuncture (13.9%).

### 3.6. Luteinizing hormone

Eleven studies^[32,34,36–44]^ reported LH, and the evidence network is typically centered around Acupuncture (Fig. 3B), which contains 12 nodes. Acupuncture was the predominant experimental group, whereas medicine served as the primary control group. A total of 66 direct or indirect comparisons were generated in the NMA, of which 7 comparisons were statistically significant, while the rest were not statistically significant, as shown in Figure 4B. The SUCRA results show that, in terms of ranking probability, RCGV has the highest cumulative probability (SUCRA: 76.6%), making it the best intervention to improve LH in patients with PCOS (Figure S1B, Supplemental Digital Content, https://links.lww.com/MD/Q404). The ranking order is as follows: Sham Acupuncture (88.2%) > RCGV (76.6%) > DEA (71.5%) > EA + Medication (66.3%) > EA (59.8%) > Control (56.8%) > CPA/EE (45.4%) > Abdominal Acupuncture (40%) > Acupuncture (39.8%) > Medication (28%) > Physical Therapist (16.7%) > Acupuncture + Medication (10.8%).

### 3.7. Luteinizing hormone/follicle-stimulating hormone (LH/FSH)

Twelve studies^[21,32,34,36–44]^reported LH/FSH, and the evidence network is typically centered around Acupuncture (Fig. 3C), with 8 nodes in the figure. Acupuncture constituted the predominant experimental group, while Physical Therapist represented the most prevalent control group. Figure 4C shows the effects of different TCM external treatments on LH/FSH, and there was no statistically significant comparison between all groups. The SUCRA results show that, in terms of ranking probability, Abdominal Acupuncture has the highest cumulative probability (SUCRA: 58.6%), making it the best intervention to improve LH/FSH in patients with PCOS (Figure S1C, Supplemental Digital Content, https://links.lww.com/MD/Q404). The ranking order is as follows: Sham Acupuncture (88%) > Abdominal Acupuncture (58.6%) > EA (54.7%) > CPA/EE (52.6%) > Acupuncture (43.9%) > Control (40.5%) > Medication (39.5%) > Physical Therapist (22.3%).

### 3.8. Testosterone

Twelve studies^[21,32–34,36–42,44]^ reported T, and the evidence network is typically centered around Acupuncture (Fig. 3D), which contains 11 nodes. Acupuncture was the most common experimental group, and medication was the most common control group. The NMA produced 66 direct or indirect comparisons, of which 23 groups were statistically significant, while the remainder were not, as illustrated in Figure 4D. The SUCRA results show that, in terms of ranking probability, Abdominal Acupuncture has the highest cumulative probability (SUCRA: 98.2%), making it the best intervention to improve T in patients with PCOS (Figure S1D, Supplemental Digital Content, https://links.lww.com/MD/Q404). The ranking order is as follows: Abdominal Acupuncture (98.2%) > EA + Medication (76.2%) > RCGV (73.3%) > Acupuncture + Medication (72%) > FNT + Medication (68.7%) > FNT (46.4%) > Medication (41.3%) > DEA (24.9%) > Acupuncture (21.3%) > Sham Acupuncture (21.1%) > CPA/EE (6.5%).

### 3.9. Body mass index

Seven studies^[21,32,34,38,41,42,44]^ reported BMI, and the evidence network is typically centered on Acupuncture (Fig. 3E), which contains 11 nodes. Acupuncture was the most common experimental group, and medication was the most common control group. The NMA produced 66 direct or indirect comparisons, of which 7 groups were statistically significant, while the remainder were not, as illustrated in Figure 4E. The SUCRA results show that, in terms of ranking probability, Acupuncture + Medication has the highest cumulative probability (SUCRA: 98.2%), making it the best intervention to improve BMI in patients with PCOS (Figure S1E, Supplemental Digital Content, https://links.lww.com/MD/Q404). The specific ranking order is as follows: Acupuncture + Medication (90.4%) > Physical Therapist (67.9%) > EA (65.6%) > Abdominal Acupuncture (58.4%) > Control (56.5%) > Acupuncture (53.1%) > Sham EA (50.3%) > Sham Acupuncture (44.7%) > CPA/EE (34.3%) > DEA (22.8%) > Medication (5.9%).

### 3.10. Adverse effects

Nine studies^[32,34,35,37,40–44]^ reported Adverse events, and the evidence network is typically centered around Acupuncture (Fig. 3F), with 9 nodes in the diagram. Acupuncture was the most common experimental group, and medication was the most common control group. Figure 4F illustrates the impact of various TCM external therapies on adverse outcomes in PCOS patients, with 7 groups demonstrating statistical significance, while the remaining groups did not achieve statistical significance. In terms of ranking probability, Abdominal Acupuncture has the highest cumulative probability (SUCRA: 96.9%), making it the treatment measure with the highest probability of causing Adverse events in patients with PCOS (Figure S1F, Supplemental Digital Content, https://links.lww.com/MD/Q404). The ranking order is as follows: Abdominal Acupuncture (96.9%) > CPA/EE (76.2%) > EA + Medication (72.2%) > Acupuncture (48.2%) > FNT (47.9%) > FNT + Medication (46.1%) > DEA (36.8%) > Medication (18.5%) > Sham Acupuncture (7.3%).

### 3.11. Publication bias

Funnel plots for 15 research were created in order to identify any potential publication bias in the included studies. Studies indicated that there was little chance of publication bias (Figure S2, Supplemental Digital Content, https://links.lww.com/MD/Q404).

### 3.12. Inconsistency test

The global and local consistency of the included studies was assessed. According to the results of the global inconsistency tests, significant inconsistency was detected for FSH (χ² = 6.62, *P* = .013), LH (χ² = 8.69, *P* = .003), and Adverse effects (χ² = 5.62, *P* = .022), whereas no significant inconsistency was found for T (χ² = 1.09, *P* = .297), BMI (χ² = 0.09, *P* = .763), and FSH–LH (χ² = 0.23, *P* = .633). A network forest plot was generated (Figure S3, Supplemental Digital Content, https://links.lww.com/MD/Q404).

## 4. Discussion

To the best of our knowledge, this is the inaugural review and NMA assessing the comparative efficacy and safety of different TCM external treatments for PCOS. In this study, NMA was performed on the 15 included studies using FSH, LH, LH/FSH, T, BMI and Adverse effects. Additionally, synthesis of evidence from 15 RCTs involving 1274 patients was conducted in order to provide more comprehensive and reliable evidence-based medical evidence for the clinical use of TCM external treatment in the treatment of PCOS.

The reproductive system is regulated by the hypothalamic-pituitary-gonadal axis under normal physiological conditions. Within this axis, pulsatile GnRH release from the hypothalamus stimulates the anterior pituitary to secrete LH and FSH, which subsequently promote steroid production by the ovaries.^[46]^ FSH facilitates follicle development, while LH triggers ovulation, with both hormones being secreted by the pituitary during the menstrual cycle.^[47]^ FSH is elevated during the follicular phase of the menstrual cycle, promoting follicular maturation and stimulating estrogen secretion, which inhibits secretion through negative feedback as follicles mature. LH rises sharply during ovulation, triggering the follicle to rupture and release the egg, and the LH level decreases after ovulation, while the corpus luteum secretes progesterone to further inhibit LH secretion. However, abnormal gonadotropin secretion patterns, characterized by elevated serum LH levels, low normal FSH concentrations, and increased LH/FSH ratios, are consistently observed in women with PCOS.^[48]^ In patients with PCOS, low FSH levels lead to incomplete follicular development and difficulty in maturation, while abnormally elevated LH levels, particularly during the follicular phase of the menstrual cycle, result in a high LH/FSH ratio, a change caused by a combination of dysregulated pituitary secretion, abnormal GnRH pulse patterns, and possible IR.^[46,49]^ In addition, high LH levels stimulate the ovaries to secrete excessive androgens, which further aggravates the symptoms of PCOS, such as hyperandrogenism, menstrual irregularities, and infertility.

In studies evaluating the improvement of FSH levels in patients with PCOS using TCM external treatments, EA may be the most effective intervention. The efficacy of EA is likely closely linked to its regulatory impact on the HPO axis. The physiological regulation of the female reproductive system involves complex interactions between neuroendocrine signaling and the HPO axis. Any factor that disrupts these connections may result in metabolic and reproductive dysfunction. PCOS, a disease closely related to HPO axis dysregulation, is characterized by follicular dysplasia, hyperandrogenism, and metabolic abnormalities.^[50,51]^ Previous studies have demonstrated that EA effectively regulates hormonal fluctuations of the HPO axis in rats by stimulating the Sanyinjiao (SP6) and Zusanli (ST36) acupuncture points. This is manifested as increased hypothalamic GnRH secretion, initial decreases followed by increases in FSH and LH levels, delayed changes in serum estradiol (E2) and progesterone, and opposite changes in serum prostaglandin E2 and norepinephrine levels, suggesting that EA may regulate neuroendocrine signals of the HPO axis to restore hormonal balance.^[52]^ Moreover, EA can enhance reproductive traits by modulating sex hormone levels and the activity of the HPO axis in PCOS rats. Specifically, EA may improve reproductive abnormalities in PCOS by attenuating androgen activity and modulating the function of the kisspeptin-GnRH/LH circuit. This mechanism may be closely associated with the downregulation of hypothalamic kisspeptin protein expression induced by EA.^[53,54]^ As a key pathway for reproductive hormone regulation, the kisspeptin-GnRH/LH axis plays a significant role in the female reproductive system, affecting follicle development, ovulation, and the normal progression of the menstrual cycle.

In conclusion, EA may serve as an effective therapeutic approach for enhancing hormone levels and reproductive function in individuals with PCOS by modulating the HPO axis and the kisspeptin-GnRH/LH axis. In evaluating the effect of TCM external treatment on the improvement of LH levels in patients with PCOS, the results indicated that RCGV (Rendumai acupuncture) was the most effective intervention. RCGV is a special TCM external acupuncture method that exerts therapeutic effects by acupuncture at specific acupuncture points on the Ren and Du pulses.^[36]^ Meta-analyses findings have demonstrated that acupuncture exerts a significant positive regulatory effect on LH levels in patients with PCOS.^[55]^ For instance, Chen et al demonstrated that acupuncture significantly reduced serum LH levels in PCOS-induced rats and inhibited the PI3K/AKT/mTOR signaling pathway by downregulating LncMEG3 expression, thereby attenuating granulosa cell autophagy and promoting the normalization of cell proliferation.^[26]^ Moreover, a separate NMA indicated that the combination of acupuncture and pharmacotherapy was more effective in improving LH levels in infertile patients with PCOS compared to pharmacotherapy alone.^[23]^ In addition, this research found that abdominal acupuncture is the best TCM external treatment to improve LH/FSH levels in PCOS patients. Wu et al^[56]^ found that acupuncture combined with clomiphene improved PCOS by reducing the LH/FSH ratio and modulating specific gut microbial genera, including decreasing *Erysipelatoclostridium* and *Proteus*, and increasing *Agathobacter*. It has also been shown that hand acupuncture can reduce LH/FSH levels in PCOS patients compared with sham acupuncture. Nonetheless, it appears contentious if various acupuncture protocols influence the monthly ovulation rate or LH/FSH levels in patients with PCOS.^[57]^ However, the effects of different acupuncture protocols on ovulation rate and LH/FSH ratio in patients with PCOS remain controversial.^[37,43]^ Nevertheless, recent meta-analyses have shown that acupuncture alone, as well as acupuncture combined with herbal medicine or moxibustion, has advantages in reducing serum LH/FSH levels in PCOS patients.^[58]^

As a specialized form of acupuncture, abdominal acupuncture has been consistently supported by meta-analyses demonstrating its ability to lower T levels in PCOS patients.^[55,59]^ Furthermore, clinical studies have indicated that acupuncture significantly reduces T levels in individuals with kidney deficiency syndromes.^[60]^ Additionally, acupuncture has been shown to regulate the HPO axis, thereby exerting beneficial effects on the female reproductive endocrine system.^[61]^ In addition, low-frequency EA reduces the secretion of β-endorphins by regulating the opioid peptide system, especially by decreasing the mRNA expression of Oprm1 and Oprk1 in the hypothalamic arc, thereby inhibiting GnRH pulse and pituitary LH release, ultimately reducing T levels in PCOS patients.^[62]^

Obesity is an important contributor to the development and exacerbation of IR,^[63,64]^ and IR is a common pathological feature observed in patients with PCOS. IR not only triggers hyperinsulinemia but also stimulates the ovaries to secrete more androgens, further aggravating PCOS symptoms such as irregular menstruation, hirsutism, and acne.^[65,66]^ Obesity exacerbates IR and induces metabolic problems by the release of pro-inflammatory cytokines and adipokines from visceral fat.^[67]^ In patients with PCOS, the adverse effects of obesity and insulin are exacerbated by abnormal expression of adipokines, especially the upregulation of leptin and resist in and the downregulation of adiponectin.^[67]^ BMI is a key factor in the management of patients with PCOS, and abdominal obesity often exacerbates pathological processes such as IR and hormone imbalance, thereby aggravating symptoms and increasing the risk of complications. Therefore, weight loss, especially abdominal fat reduction, has a significant therapeutic effect for patients with PCOS. In this research, through an in-depth analysis of external treatment to improve BMI in PCOS patients, it was found that “acupuncture + drug therapy” was the best intervention. Acupuncture is widely used in complementary and alternative medicine for weight loss. In a previous meta-analysis of acupuncture and related therapies for obesity, acupuncture and related treatments demonstrated that acupuncture and related treatments are the best way to reduce body weight and BMI.^[68]^ Acupuncture has demonstrated efficacy in treating obesity by modulating the arcuate nucleus of the hypothalamus and its associated neural pathways, enhancing the expression of appetite-suppressing neuropeptides such as α-MSH and CART, thereby reducing food intake and body weight.^[69]^ Moreover, acupuncture significantly lowers fasting insulin, homeostasis model assessment of IR, and waist circumference in patients with PCOS and abdominal obesity, resulting in a more pronounced reduction in IR compared to diet and exercise interventions alone.^[70]^ The “dialing needling method” has also been shown to significantly decrease body weight and ovarian volume in patients with abdominal obesity and PCOS, with its therapeutic effects potentially related to alterations in fasting insulin, LH, and T levels.^[71]^

Limitations of this study: First, the overall methodological quality of the included studies was relatively low, which may introduce publication bias. Several studies did not clearly report the methods of randomization, lacked trial registration, and provided no information on allocation concealment or blinding procedures, thereby limiting the reliability and internal validity of the findings. Second, the number of included studies was limited, and the outcome indicators selected in this analysis did not comprehensively cover all intervention modalities, potentially reducing the statistical power and generalizability of the results. To validate the findings of this study, additional RCTs with large sample sizes, high quality, and objective, detailed reporting of adverse responses covering more external TCM therapies are required. Third, indirect comparisons versus NMA included multiple different trials with different treatment comparison types, and effect-influencing factors were present comparisons, leading to certain limitations in this study. Fourth, the specific methods of acupuncture and the selection of acupoints were not clearly defined in the included studies. Therefore, the term “TCM external treatment” used in this study represents a broad conceptual framework rather than a standardized or uniform therapeutic protocol.

## 5. Conclusion

The external treatment approach of TCM offers advantages such as ease of implementation, significant therapeutic efficacy, and favorable economic benefits. This study validates the clinical effectiveness of different TCM external therapies for PCOS and compares the outcomes of various TCM external treatment methods. It was concluded that EA may be an effective intervention to improve the FSH level of PCOS patients, and RCGV is the most effective intervention to improve the LH level of PCOS patients. Abdominal acupuncture is the most effective intervention to improve LH/FSH and T levels in patients with PCOS. “Acupuncture + drug therapy” is the best intervention for BMI in patients with PCOS. However, the safety of abdominal acupuncture has been questioned and needs to be carefully considered in clinical studies. This study establishes a precise clinical basis for the entire treatment of PCOS. It is hoped that this treatment regimen can be widely promoted in clinical practice and ultimately benefit PCOS patients.

## Author contributions

**Conceptualization:** Jiahui Ye, Hua Guo, Hanmei Lin.

**Data curation:** Jiahui Ye, Hong Su.

**Formal analysis:** Jiahui Ye, Qianru Zeng, Qilin Jin, Hanmei Lin.

**Funding acquisition:** Hua Guo, Hong Su.

**Investigation:** Jiahui Ye, Qianru Zeng, Ziqing Gan, Qilin Jin, Hanmei Lin.

**Methodology:** Hua Guo, Ziqing Gan, Hong Su.

**Project administration:** Ziqing Gan, Hong Su.

**Resources:** Qianru Zeng, Hanmei Lin.

**Software:** Hua Guo, Qianru Zeng, Ziqing Gan, Qilin Jin.

**Supervision:** Qianru Zeng, Ziqing Gan, Qilin Jin, Hong Su, Hanmei Lin.

**Validation:** Qianru Zeng, Qilin Jin, Hong Su.

**Visualization:** Jiahui Ye, Hua Guo, Qilin Jin, Hong Su, Hanmei Lin.

**Writing – original draft:** Jiahui Ye.

**Writing – review & editing:** Hanmei Lin.

## Supplementary Material


