# Supplementary material for: Comparative efficacy and safety of different traditional Chinese medicine external therapies for polycystic ovary syndrome in women: A network meta-analysis
Source: Medicine (Baltimore). 2025 Oct 24;104(43):e44441. doi: 10.1097/MD.0000000000044441 (PMC12558321; doi:10.1097/MD.0000000000044441)
Supplement: Supplementary file 1 [file medi-104-e44441-s001.pdf]

Supplementary Figure 1. Sucra rank

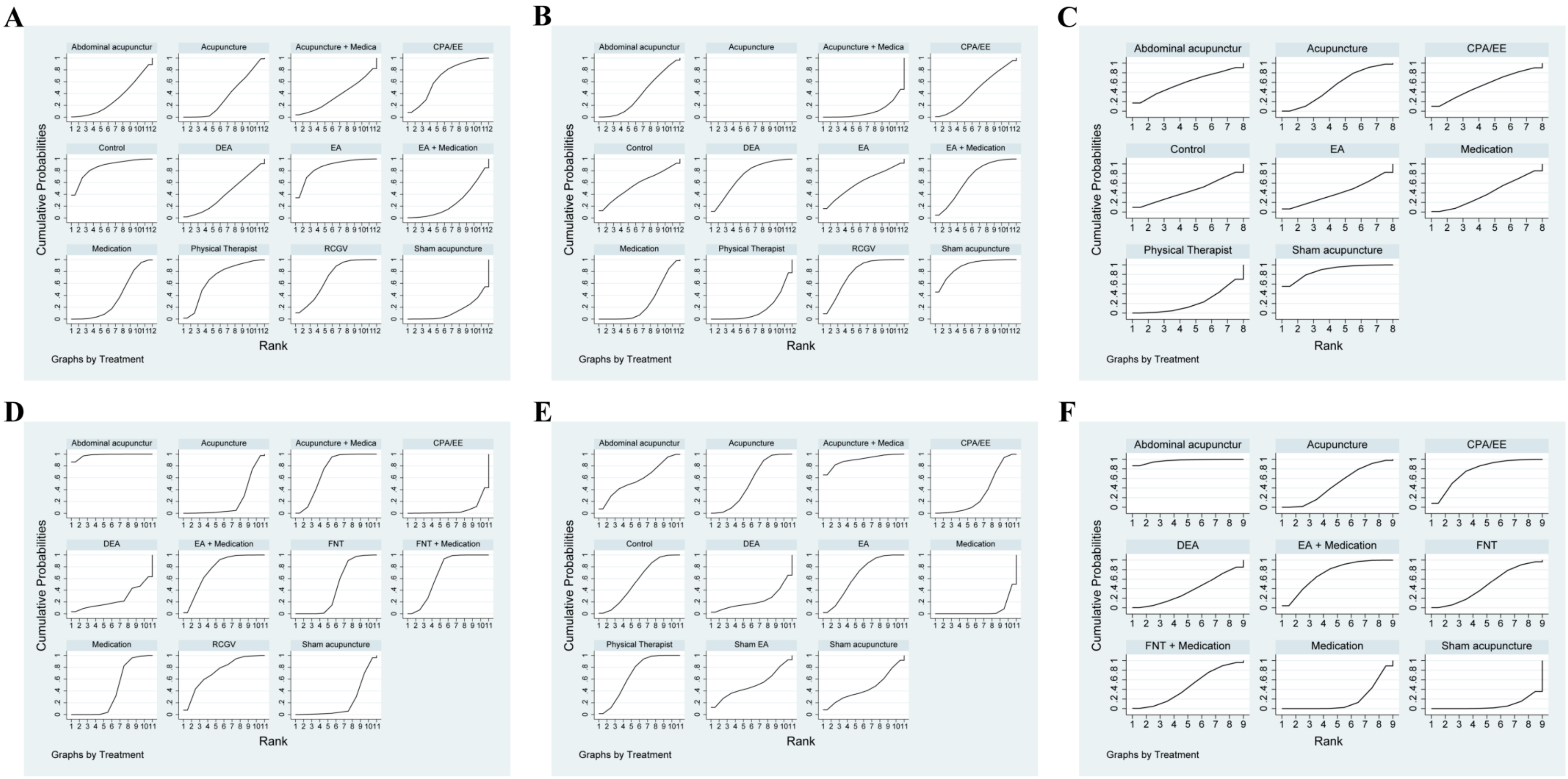

Note： A:FSH;B:LH; C:LH/FSH; D:T ;E:BMI ;F:Adverse

Supplementary Figure 2. Funnel plot.

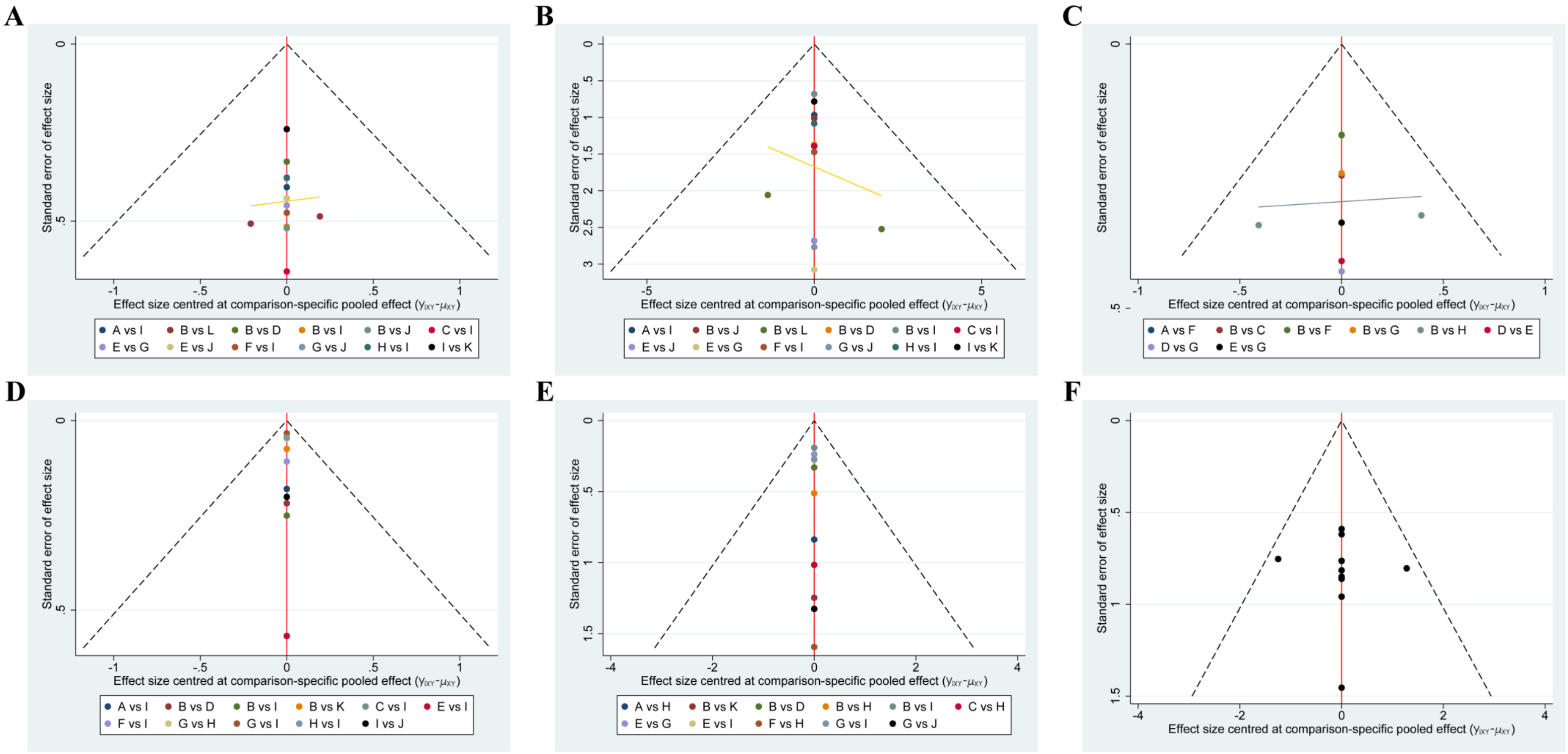

Note： A:FSH;B:LH; C:LH/FSH ; D:T ;E:BMI ;F:Adverse

Supplementary Figure 3. consistency forest

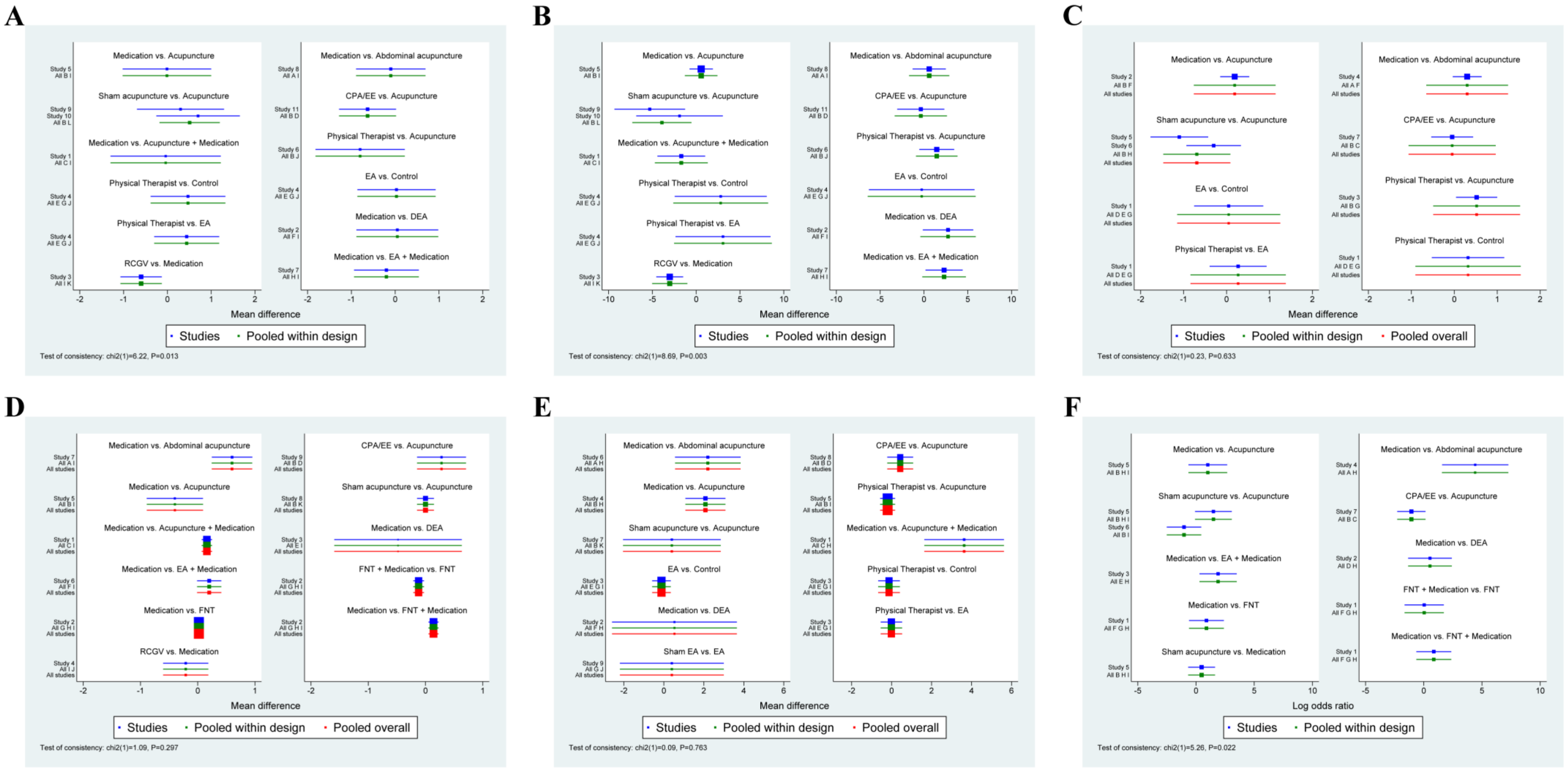

Note： A:FSH;B:LH; C:LH/FSH; D:T ;E:BMI ;F:Adverse
